# Family-to-work conflict linked to psychological distress and family life satisfaction during the second year of the COVID-19 pandemic in dual-earner parents with adolescents

**DOI:** 10.3389/fpubh.2024.1476549

**Published:** 2024-11-29

**Authors:** Berta Schnettler, Edgardo Miranda-Zapata, Ligia Orellana, Mahia Saracostti, Héctor Poblete, Andrés Concha-Salgado, Germán Lobos, Cristian Adasme-Berríos, María Lapo, Katherine Beroiza, Leonor Riquelme-Segura, José A. Sepúlveda, Enid Thomas

**Affiliations:** ^1^Facultad de Ciencias Agropecuarias y Medioambiente, Universidad de La Frontera, Temuco, Chile; ^2^Centro de Excelencia en Psicología Económica y del Consumo, Universidad de La Frontera, Temuco, Chile; ^3^Scientific and Technological Bioresource Nucleus (BIOREN-UFRO), Universidad de La Frontera, Temuco, Chile; ^4^Universidad Católica de Santiago de Guayaquil, Guayaquil, Ecuador; ^5^Universidad Autónoma de Chile, Temuco, Chile; ^6^Departamento de Psicología, Universidad de La Frontera, Temuco, Chile; ^7^Escuela de Trabajo Social, Universidad de Valparaíso, Valparaíso, Chile; ^8^Departamento de Trabajo Social, Universidad de Chile, Santiago, Chile; ^9^Facultad de Economía y Negocios, Universidad de Talca, Talca, Chile; ^10^Departamento de Economía y Administración, Universidad Católica del Maule, Talca, Chile; ^11^Departamento de Trabajo Social, Universidad de La Frontera, Temuco, Chile; ^12^Doctorado en Ciencias Agroalimentarias y Medioambiente, Universidad de La Frontera, Temuco, Chile

**Keywords:** family-to-work conflict, psychological distress, family satisfaction, dual-earner parents, adolescents, COVID-19

## Abstract

**Introduction:**

Research on work-family dynamics during the COVID-19 pandemic shows that family demands increased for workers, particularly those with children. This heightened family-to-work conflict negatively affects the subjective well-being of parents and their children. However, these outcomes have been mainly examined on individuals without considering the family as the unit of analysis. This study aimed to explore the relationships between family-to-work conflict, psychological distress, and family life satisfaction in dual-earner parents with adolescent children during the second year of the COVID-19 pandemic. Moreover, the potential mediating role of psychological distress among FtoWC and family life satisfaction and the moderating role of household monthly income were tested.

**Methods:**

The non-probabilistic sample in Chile comprised 860 dual-earner parents and one of their adolescent children (mean age 13.5 years, 50.8% female). Parents answered an online questionnaire with FtoWC scale, whereas parents and adolescents answered the Depression, Anxiety, Stress Scale and the Satisfaction with Family Life Scale. Analysis was conducted using structural equation modeling and the mediation actor-partner interdependence model.

**Results:**

FtoWC was directly linked to lower family life satisfaction in fathers and via a mediating role of psychological distress in both parents. Mothers’ FtoWC was related via the mediating role of the fathers’ psychological distress to fathers’ lower family life satisfaction and via the mediating role of the adolescents’ psychological distress to adolescents’ lower family life satisfaction. Monthly household income moderated three APIM model paths involving mothers’ variables.

**Discussion:**

These findings underscore the importance of implementing family-oriented workplace policies. Such policies may help mitigate both parents’ experiences of FtoWC and their resulting psychological distress.

## 1 Introduction

Workers’ difficulties balancing work and family obligations increased with the COVID-19 pandemic (1, 2). The conflict between the work and family domains is termed work–family conflict, in which work and family roles seem mutually incompatible (3). The literature shows that work–family conflicts have increased (4, 5) since the start of the pandemic in March 2020 (6). However, several authors have highlighted a more significant increase in family-to-work conflict (FtoWC)—how family characteristics impact the workplace—than that of work-to-family conflict (WtoFC)—how work-related factors influence the family domain—(4, 5, 7). Studies show that, compared to pre-pandemic times, parents have faced new and more challenging responsibilities in the family domain after the outbreak (8), which have significantly affected family life (9, 10). Lockdown measures forced family members to stay home, increasing workers’ family demands such as taking on childcare and home-schooling duties and doing multiple household chores. Furthermore, these additional family tasks and responsibilities have been associated with deteriorated mental health (1, 9).

On this basis, we sought to explore the complexities of family-to-work conflict during the second year of the pandemic. To this end, this study adopts the matching hypothesis (11). According to this hypothesis, the primary effects of conflict (FtoWC for this study) manifest predominantly in the domain where the conflict originates, in this case, the family. Additionally, our study draws on the Conservation of Resources (COR) theory (12) because demands deplete limited resources, such as time, energy, and psychological resources, as individuals navigate the challenges of everyday life (13).

Work–family conflict hampers individuals’ mental health and well-being, and those of others close to them, such as their spouses and children (2, 14, 15). For instance, research shows that work–family conflict can be a source of stress for dual-earner couples, particularly during the COVID-19 pandemic (1, 2). Furthermore, for workers with children, the demands stemming from parent–child dynamics also increased during the pandemic, with specific difficulties depending on the children’s developmental stage. On their part, adolescents faced heightened vulnerabilities in their mental health as their developmental trajectory was disrupted (16). Thus, parents have struggled to balance their work and respond to their adolescent children’s needs (17).

Work–family conflict research has rarely included the workers’ adolescent children, a group at increased risk for mental health problems (16). Earlier studies have emphasized the stress of rearing younger children, but more recent studies have explored parents’ well-being when their children are older (18). For instance, mothers and fathers of adolescents report lower happiness scores when spending time with their children than parents of infants or toddlers (19). Moreover, most studies on the work-family interface and children’s mental health and well-being have been conducted with samples of US and Australian children, mainly from the standpoint of WtoFC or work–family conflict. The current literature also tends to focus on mothers’ work-family interface, which results in a gap in understanding the differential effects of work-family interactions on children for mothers and fathers (14). However, knowledge of the underlying mechanisms that explain the association between FtoWC and family satisfaction is sparse, given that research has focused on this type of conflict following the work-to-family direction (20, 21), neglecting the interdependency between family members (2, 14). Furthermore, the knowledge about partner associations of parental FtoWC and spouses’ outcomes during the COVID-19 pandemic is still limited (8).

However, our recent findings in the context of dual-earner families in Chile during the first year of the pandemic indicate that job satisfaction is a mediator between FtoWC and family satisfaction for both mothers and fathers. We discovered a negative association between FtoWC and family satisfaction for both parents (22). Moreover, our research revealed that higher FtoWC is correlated with more pronounced psychological distress and lower job satisfaction in both parents (23). Therefore, in this study, we sought to revisit the relationship between parents’ FtoWC and psychological distress during the second year of the pandemic and to provide novel insights into the influence of these variables on parents’ and one adolescent child’s satisfaction with family life. For this purpose, the data was examined using the mediation Actor-Partner Interdependence Model (APIM) framework (24), which evaluates how the effects of an individual and their partner may be influenced by the characteristics of the individuals in the dyad and their interactions (25). Lastly, considering that families navigate the pandemic with different financial situations (9, 26), we also explored the moderating role of household monthly income.

### 1.1 Relationships between family-to-work conflict, family life satisfaction, and psychological distress

The COR theory states that individuals seek to obtain, retain, and protect valuable things, known as resources, which help them deal with stressors (12). When individuals’ efforts to protect personal resources fail, psychological distress may occur, for instance, when employees face high demands in the family domain (15, 27). In this sense, COR theory also proposes a loss spiral that shows how stress can further deplete resources such as energy and positive moods (12). We can characterize the relationships between FtoWC and psychological distress as a resource loss spiral that may negatively affect family satisfaction.

**Figure 1:**
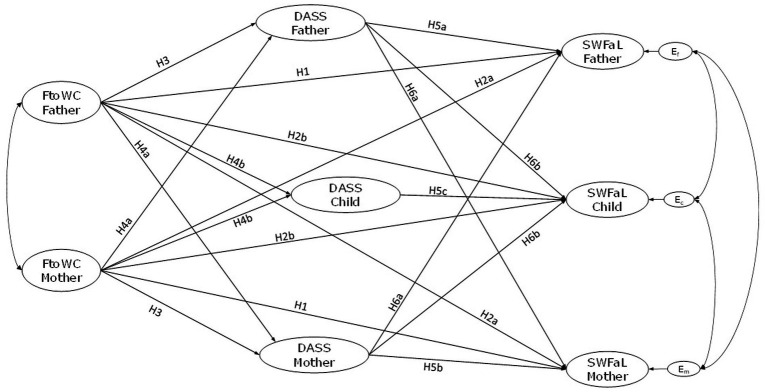
Conceptual model of the proposed actor and partner effects between family-to-work conflict (FtoWC), psychological distress (DASS), and satisfaction with family life (SWFaL) in dual-earner parents with adolescent children. E_m_, E_f_, and E_c_: residual errors on SWFaL for mothers, fathers, and adolescent children, respectively. The indirect effects of Psychological Distress (H7) shown in the conceptual path diagram to avoid cluttering the figure.

Exploring the impact of FtoWC in the family domain can be aided by the construct of satisfaction with family life (family satisfaction from here on in). This construct is defined as individuals’ subjective, conscious assessment of their family life (28). The matching hypothesis might explain this potential link between FtoWC and family satisfaction (11). The matching hypothesis states that the domain in which work–family conflict originates suffers the primary effects of this conflict. In this stance, if workers’ family commitments (e.g., caring for others, conflicts with family members) adversely affect their work quality or productivity, career, and aspirations (29), workers might become angry or resentful against their family, and thus experience lower family satisfaction (11). Recent studies have provided evidence of the matching hypothesis regarding the negative association between FtoWC and family satisfaction at an individual level (11, 20–23, 30, 31). Thus, we expect that FtoWC and family satisfaction will show a negative relationship for both parents and posit the first hypothesis (Figure 1):

*H*1. Family-to-work conflict is negatively associated with family satisfaction for each parent (actor effects).

The strain resulting from FtoWC in the individual can also cross over between different family members (32, 33). During the first year of the COVID-19 pandemic, Orellana, Schnettler et al. (22) found that fathers’ FtoWC negatively crossed over to the mothers’ family satisfaction. In parent–child relationships, studies show that parent’s WtoFC harms adolescent children’s domain satisfaction via negative parent–child relationships (17, 34, 35). Moreover, Leach et al. (36) found that both WtoFC and FtoWC helped shape the quality of parent–child relationships that influence children’s well-being. Hence, we expect one parent’s FtoWC to cross over to the family satisfaction of the other parent and their adolescent child. The following hypothesis is thus proposed for mothers, fathers, and adolescents (Figure 1):

*H*2. Family-to-work conflict of one parent is negatively associated with (a) the other parent’s and (b) the adolescent’s family satisfaction (partner effects).

Previous research shows that FtoWC may lead to negative emotions (8, 9, 27, 37). Excessive family demands and responsibilities related to FtoWC may entail time constraints (38) and stress, which ultimately could lead to mental health difficulties (7, 37). In this regard, some studies during the COVID-19 pandemic have associated FtoWC with increased psychological distress in employees who belong to information technology and information technology-enabled services, financial services, and education sector in India, in workers from diverse occupations and job sectors in Canada and Chile (23, 31, 39, 40); depression in female social workers from China (27); and anxiety and depression in workers from diverse occupations and job sectors in Argentina (9). Thus, we expect that FtoWC and psychological distress will show a positive relationship for both parents and posit the following hypothesis (Figure 1):

*H*3. Family-to-work conflict is positively associated with psychological distress for each parent (actor effects).

The evidence of crossover effects from one individual’s FtoWC to their partner’s mental health is sparse and mixed (41). However, these effects could be expected following the COR theory, which posits that one individual’s resource loss can entail a threat or actual loss of resources in their partner (41). Some pre-pandemic studies support this assumption, linking higher FtoWC and mental health in German different-sex dual-earner couples (42, 43). Likewise, during the first year of the pandemic in Chile, Schnettler et al. (23) reported that mothers’ FtoWC crossed over to fathers, increasing their psychological distress. However, also during the COVID-19 pandemic in China, with a sample of dual-earner parents with at least one child aged 7–18 years old, Zou et al. (8) found that both parents’ FtoWC was directly related to their depressive symptoms. However, no crossover effects between spouses were found.

We found no available studies that assessed the crossover effects of parents’ FtoWC on their adolescent children’s mental health. However, a systematic review by Bilodeau et al. (14) suggests stress (e.g., high FtoWC) in the context of COVID-19 in parents can cross over to their children, negatively affecting their behaviors or mental health. Similarly, pre-pandemic longitudinal studies conducted in Australia have shown that parental work–family conflict is associated with negative parent–child interactions and mental health outcomes in children (44–46). However, these studies used a single measure that captured WtoFC and FtoWC. Another longitudinal study in Australia by Leach et al. (36), also using a single work–family conflict measure that assessed it in both directions, found that work–family conflict is a stressor that can affect all family members (36). Furthermore, these authors did not find differences by the parents’ gender, therefore suggesting that mothers and fathers can experience the impact of work–family conflict and its associated negative outcomes. Based on these findings, we propose the following hypothesis (Figure 1):

*H*4. Family-to-work conflict of one parent is positively associated with (a) the other parent’s and (b) the adolescent’s psychological distress (partner effects).

FtoWC and psychological distress can set off a resource loss spiral, as proposed in the COR (13). Psychological distress has also been shown to be negatively related to family satisfaction in adults (31, 40, 47) and adolescents (48–50) at an individual level. Thus, based on this theoretical and empirical background, we posit the following hypothesis (Figure 1):

*H*5. Psychological distress is negatively associated with family satisfaction for (a) fathers, (b) mothers, and (c) adolescents (actor effects).

Other studies suggest this resource loss can also crossover to family members (15). For instance, individuals’ mental health can affect their partners’ well-being (51, 52), and depressive symptoms can negatively affect family interactions (47). Studies by Schnettler et al. (52) and Orellana, García et al. (51) in different-sex dual-earner couples found that depression symptoms were negatively associated with their own and with their partner’s family satisfaction.

Additional research suggests that parents’ mental health is also linked to family satisfaction levels reported by their children. Studies report links between family strain and depression and anxiety (53), and family interactions and depressive symptoms and stress (54) in adults and their children. Orellana, Schnettler et al. (32) distinguished family satisfaction profiles of mother–father-adolescent triads and reported that families with high depression, anxiety, and stress scores in parents also had lower levels of family satisfaction. This negative association between parents’ mental health and family members’ family satisfaction may be more pronounced during the COVID-19 pandemic. For instance, at the start of the pandemic in Germany, Ebert and Steinert (55) found that increasing parental depression/anxiety was significantly related to marital conflict and child maltreatment, which in turn may negatively influence family life satisfaction for all family members. On this basis, we hypothesize that one parent’s psychological distress is associated with lower family satisfaction of the other parent and their adolescent child. Therefore, we propose the following hypothesis (Figure 1):

*H*6. Psychological distress of one parent is negatively associated with (a) the other parent’s and (b) the adolescent’s family satisfaction (partner effects).

Psychological distress may be an underlying mechanism that explains the negative association between FtoWC and family satisfaction. Following the matching hypothesis, we propose that FtoWC generates psychosocial distress, which generates negative affective responses toward the family domain. For instance, Riquelme-Segura et al. (31) found that psychological distress negatively mediates between FtoWC and family satisfaction in a sample of working mothers during the COVID-19 pandemic in Chile. Also, during the first year of the pandemic in Chile, Schnettler et al. (23) reported that psychological distress mediates between FtoWC and job satisfaction at intra- and interindividual levels in dual-earner couples. In parent–child dyads, Reimann, Schulz, et al. (21) have highlighted that family relationships and environment, and parenting characteristics and styles have a negative mediating role between parents’ work–family conflict and child well-being (46, 56). Furthermore, in Germany, van den Eynde et al. (57) found that parents’ well-being is mediating in the relationship between parents’ FtoWC and child behavior. Based on the findings from these studies, which demonstrate crossover effects between individuals’ mental health problems and family-related variables of their family members (51, 52), we expect that psychological distress may have an inter-individual (i.e., crossover) mediating role. We thus propose the last hypothesis (Figure 1):

*H*7. Psychological distress has a mediating role between parents’ family-to-work conflict and satisfaction with family life for the three family members (actor and partner effects).

Lastly, research conducted in China, Argentina, Taiwan, and Thailand during the pandemic and a review study revealed a correlation between financial adversities (mainly income loss and self-reported financial problems) and heightened instances of depression, anxiety, perceived stress, FtoWC, familial conflicts, and diminished overall well-being (2, 9, 26, 58–60). Similar situations were also observed in Chile. During the COVID-19 pandemic, 59.4% of Chilean households saw a decline in their overall income. Within this group, 44.6% indicated that their income had dropped by half or more than pre-crisis levels. Pre-pandemic, 16.5% of households found their income insufficient to cover expenses; however, this figure surged to 48.8% during the pandemic. Moreover, 53.7% of households resorted to selling assets, using up savings, or disposing of property. Furthermore, 40% of households incurred debt through loans, credits, or assistance from financial institutions, family, friends, or credit facilities. The impact of the pandemic on households’ quality of life was substantial: 65.6% postponed health treatments, 19.4% struggled with food insecurity, and 21.4% faced moderate to severe anxiety or depression (61). Based on these antecedents, we posed one research question:

RQ1. Does the household monthly income moderate the associations posited in Hypotheses 1 to 6?

## 2 Methods

### 2.1 Sample and procedure

Using non-probability sampling, 430 dual-earner parents (married or cohabiting) with at least one child aged between 10 and 15 years were recruited in Temuco City, Chile (Table 1). The age range for children in this study was established following the World Health Organization’s (62) criterion of adolescence as a period between 10 and 19 years old.

**Table 1:** Sample characteristics of participant families.

Characteristic	(*n* = 430)
Age [Mean (*SD*)]	
Mother	39.6 (7.2)
Father	42.7 (8.5)
Adolescent	13.5 (2.0)
Adolescents’ gender (%)	
Male	51.9
Female	48.1
Number of family members [Mean (*SD*)]	4.4 (1.6)
Number of children [Mean (*SD*)]	2.2 (0.9)
Socioeconomic status (%)	
High	0.7
Middle	80.2
Low	19.1
Mothers’ type of employment (%)	
Employee	66.7
Self-employed	33.3
Fathers’ type of employment (%)	
Employee	72.6
Self-employed	27.4
Mothers’ working hours (%)	
45 h per week	51.4
Less than 45 h per week	48.6
Fathers’ working hours (%)	
45 h per week	74.2
Less than 45 h per week	25.8
Mothers’ place of working (%)	
Remote	26.3
Face-to-face	73.7
Fathers’ place of working (%)	
Remote	8.4
Face-to-face	91.6

The vaccination campaign against COVID-19 in Chile was initiated in January 2021, resulting in a gradual relaxation of mandatory confinement measures in various cities. By June 2021, 80% of adults had received their first vaccine dose. Temuco remained unrestricted during the data collection period. Notably, in locked-down areas, organizations were authorized to issue work permits to their employees, leading to a significant return to in-person work for many individuals (63).

Families were recruited via schools serving diverse socioeconomic populations. Trained interviewers contacted parents and provided information on the study’s aims and method and the anonymous treatment of their responses. All three family members who agreed to participate were assigned an interviewer. One family member, most often the mother, remained in contact with the interviewer through telephone and email. Families answered the questionnaires online, receiving one link per family member via e-mail. Interviewers emailed these links to their assigned families between August and December 2021. After completing the three surveys, families received a gift card worth 15 USD in Chilean pesos. This study belongs to a broader research project on the work-family interface and food-related life in Chilean dual-earner families.

Before this procedure, a pilot test was conducted with 50 families from Temuco. The method and instrument were deemed satisfactory, and no changes were made to data collection. This study was approved by the Ethics Committee of the Universidad de La Frontera (protocol 007/19).

### 2.2 Measures

#### 2.2.1 Family-to-work conflict

The four-item FtoWC measure used in this study, adapted by Kinnunen et al. (64), examines negative spillover from family to work (example item: “Your home life prevents you from spending the desired amount of time on job- or career-related activities?”). Response options are presented on a five-point scale, from 1: never to 5: very often. The validated Spanish version of this measure was used (65). In this study, all standardized factor loadings were significant (*p* < 0.001) and ranged from 0.707 to 0.975 for mothers and from 0.707 to 0.966 for fathers. The average extracted variance (AVE) values were higher than 0.50 (mothers = 0.79, fathers = 70). Internal reliability was measured with the Omega coefficient, with values of 0.93 for mothers and 0.90 for fathers.

#### 2.2.2 Psychological distress

The measure used to assess psychological distress was the 21-item Depression, Anxiety, and Stress Scale (DASS-21) (66). This scale encompasses three dimensions of distress at a subclinical level: depression (example item: “I could not seem to experience any positive feeling”), stress (example item: “I experienced breathing difficulty”), and anxiety (example item: “I found it hard to wind down”). The four-point response scale ranges from 0: it does not describe anything that happened to me, or I felt during the week; 3: yes, this happened to me a lot, or almost always. In this study, a discriminant validity analysis showed a unidimensional scale, as reported by other studies (67). This construct was termed psychological distress. This scale was validated in Chilean adults and adolescents (51, 68). In this study, the standardized factor loadings were significant (*p* < 0.001), ranging from 0.620 to 0.912 for mothers, from 0.746 to 0.928 for fathers, and from 0.733 to 0.856 for adolescents. The AVE values were good (mothers = 0.71, fathers = 0.72, adolescents = 0.76). Omega coefficients show good reliability for all three family members (mothers = 0.93; fathers = 0.94; adolescents = 0.95).

#### 2.2.3 Family satisfaction

The Satisfaction with Family Life Scale (SWFaL) (28) was used to measure this construct. This five-item scale is an adaptation of the Satisfaction with Life Scale (69), in which the word “life” is changed to “family life” (example item: “In most ways my family life is close to my ideal”). The Spanish version of this scale was used in this study, which has shown good internal consistency in Chilean adults and adolescents (10, 70). Responses are rated on a six-point Likert-type scale from 1: completely disagree to 6: completely agree. The standardized factor loadings of this scale were statistically significant (*p* < 0.001) in this study and ranged: For mothers, from 0.722 to 0.917; for fathers, from 0.824 to 0.918; and for adolescents, from 0.809 to 920. The AVE values were good (mothers = 0.72, fathers = 0.78, adolescents = 0.76), and good reliability was found for the three family members’ responses (mothers = 0.93; fathers = 0.93; adolescents = 0.94).

#### 2.2.4 Socioeconomic status

Other questions enquired about the three family members’ age. Parents were asked about their type of employment, the number of working hours per week, where they worked, and their monthly income. Mothers were asked about the number of family members and children in the home. Family socioeconomic status (SES) was obtained by combining ranges of the household monthly income and the number of family members in a matrix (71).

### 2.3 Data analysis

The Statistical Package of Social Sciences (SPSS) v.23 was used for descriptive analysis. The first seven hypotheses were tested following Weidmann et al. (72) and Schnettler, Miranda-Zapata et al. (35). In this regard, we conducted an extended version of the mediation actor-partner interdependence model (APIM, (24)). Namely, we used a triadic APIM approach using SEM with distinguishable triads conducted in Mplus 8.4. Actor effects (i.e., individual effects) are those associations between one parent’s FtoWC, DASS, and SWFaL, whereas partner (i.e., interpersonal) effects are those associations between FtoWC, DASS, and SWFaL for mother, father, and adolescent. Partner effects are dyadic associations within the dyads mother–father and parent-adolescent, but the overall approach of this study examines triadic effects.

Following Kenny et al. (24), sources of interdependence between partners are controlled by specifying correlations between the residual errors of the dependent variables (DASS and SWFaL) of the three family members and between both parents’ FtoWC. The model incorporated other variables to control for, such as parents’ and adolescents’ ages, parents’ type of employment, number of working hours, the place where they worked, family SES, and the number of children.

The Structural Equation Modeling (SEM) analysis utilized the weighted least square mean and variance adjusted (WLSMV) method to estimate the parameters of the structural model. The study employed the polychoric correlation matrix to accommodate the ordinal nature of the items. Model fit assessment was performed using the following criteria: The Tucker-Lewis index (TLI) and the comparative fit index (CFI), both of which indicate a favorable fit when surpassing a threshold of 0.95; additionally, the root mean square error of approximation (RMSEA) suggests a strong fit when values are below 0.06, according to Hu and Bentler (73).

For hypothesis 7, we investigated whether the psychological distress experienced by each family member acted as a mediator in the relationship between both parents’ FtoWC and the three members’ family satisfaction. To assess whether psychological distress played a mediating role between the independent and dependent variables, we conducted SEM with a bias-corrected (BC) bootstrap confidence interval approach, using 1,000 samples as recommended by Lau and Cheung (74). A mediating role is proposed when the BC confidence intervals do not include zero.

Finally, we examined the moderating effect suggested in RQ1 using multi-group SEM (75). This analysis involved comparing direct effect parameters between groups for each model path, as defined by dichotomous moderators. We identified evidence of a moderation effect when there was a statistically significant difference in a direct estimate within the model between groups.

## 3 Results

### 3.1 Sample description

The sample comprised 430 dual-earner parents with at least one adolescent aged between 10 and 15. Table 1 displays the sociodemographic characteristics of the sample.

Table 2 shows the average scores and correlations for family-to-work conflict (FtoWC), psychological distress (DASS), and family (SWFaL). Most correlations showed significance and aligned with the expected outcome, except the correlation between mothers’ FtoWC and adolescents’ SWFaL, between mothers’ DASS and adolescents’ SWFaL, and between adolescents’ DASS and mothers’ SWFaL. Mothers scored significantly higher than fathers in FtoWC (*t* = 2.829, *p* = 0.005). Mothers and adolescents did not differ in the average scores for DASS; their scores were significantly higher than the fathers’ (*F* = 13.420, *p* < 0.001). Mothers scored considerably lower than fathers and adolescents in the SWFaL (*F* = 6.451, *p* = 0.002).

**Table 2:** Descriptive statistics and correlations for family-to-work conflict (FtoWC), psychological distress (DASS), and satisfaction with family life (SWFaL) in different-sex dual-earner parents with adolescent children.

		Correlations							
	M (SD)	1	2	3	4	5	6	7	8
1. Mothers’ FtoWC	7.52 (3.24)	1	0.443**	0.388**	0.223**	0.139**	−0.170**	−0.157**	0.029**
2. Fathers’ FtoWC	6.93 (2.91)		1	0.242**	0.298**	0.130**	−0.131**	−0.225**	−0.103*
3. Mothers’ DASS	29.97 (10.34)			1	0.445**	0.294**	−0.263**	−0.184**	−0.094
4. Fathers’ DASS	27.13 (8.52)				1	0.348 **	−0.165**	−0.330**	−0.186**
5. Adolescent’s DASS	29.21 (10.44)					1	−0.050	−0.138**	−0.424**
6. Mothers’ SWFaL	23.29 (5.00)						1	0.418**	0.236**
7. Fathers’ SWFaL	24.45 (4.74)							1	0.400**
8. Adolescents’ SWFaL	24.94 (4.81)								1

*p < 0.05, two-tailed. **p < 0.01, two-tailed.

### 3.2 Actor and partner effects

**Figure 2:**
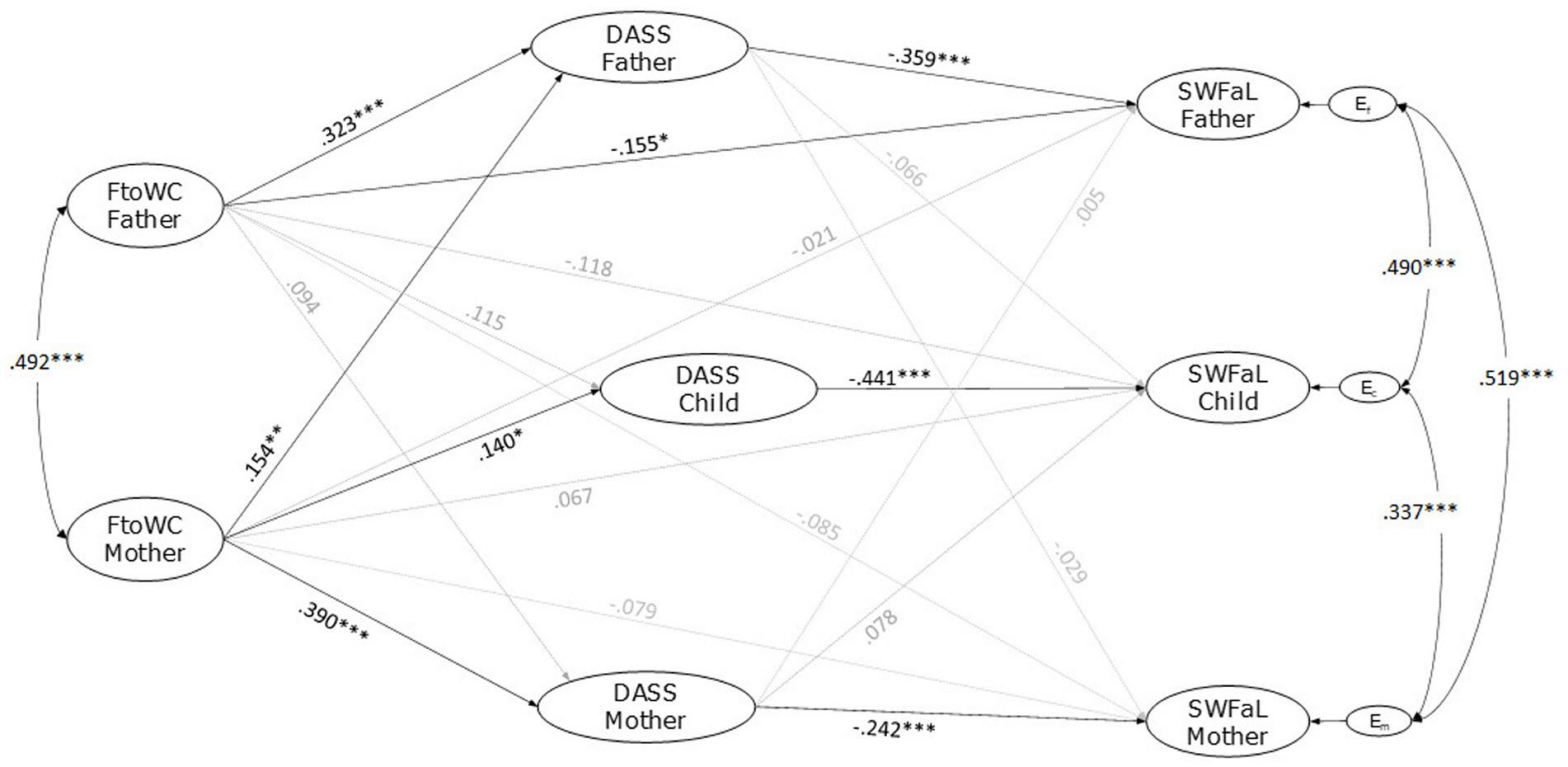
Actor-partner interdependence model of the effect family-to-work conflict (FtoWC), psychological distress (DASS), and satisfaction with family life (SWFaL) in dual-earner parents with adolescent children. E_m_, E_f_, and E_c_: residual errors on SWFaL for mothers, fathers, and adolescent children, respectively. The control for the effects of the three family members age, parents type of employment, and their number of working hours as well as the family SES and the number of children on the dependent variables of the three family member (DASS and SWFaL), were not shown in the path diagram. *p < 0.05, **p < 0.01, ***p < 0.001.

Figure 2 shows the estimation of the APIM model. This model assessing the mother’s and father’s family-to-work conflict (FtoWC) and the three family members’ psychological distress (DASS) and satisfaction with family life (SWFaL) had a good fit with the data (CFI = 0.978, TLI = 0.976, RMSEA = 0.024). Both parents’ FtoWC showed a significant correlation (covariance, *r* = 0.492, *p* < 0.001). Other significant correlations showed between the residual errors of mothers’ and fathers’ DASS (*r* = 0.490, *p* < 0.001), mothers’ and adolescents’ DASS (*r* = 0.338, *p* < 0.001), and between fathers’ and adolescents’ DASS (*r* = 0.385, *p* < 0.001). Likewise, significant correlations were found between the residual errors of mothers’ and fathers’ SWFaL (*r* = 0.519, *p* < 0.001), mothers’ and adolescents’ SWFaL (*r* = 0.337, *p* < 0.001), and between fathers’ and adolescents’ SWFaL (*r* = 0.490, *p* < 0.001).

H1 proposed that FtoWC is negatively associated with SWFaL for each parent. Whereas the standardized path coefficients indicated that fathers’ FtoWC was negatively related to their SWFaL (*γ* = −0.155, *p* = 0.011), mothers’ FtoWC (γ = −0.079, *p* = 0.197) was not; thus, H1 was supported only for fathers.

H2 sought partner effects, namely, that FtoWC of one parent is negatively associated with the other parent’s (H2a) and with the adolescent’s (H2b) SWFaL. Neither fathers’ FtoWC was significantly associated with mothers’ SWFaL (*γ* = −0.085, *p* = 0.170), nor mothers’ FtoWC was significantly associated with fathers’ SWFaL (γ = −0.021, *p* = 0.713). These findings did not support H2a. Similarly, neither mothers’ (*γ* = 0.067, *p* = 0.301) nor fathers’ (*γ* = −0.118, *p* = 0.064) FtoWC was statistically associated with adolescents’ SWFaL. H2b was not supported.

H3 stated that FtoWC is positively associated with DASS for each parent. Fathers’ (γ = 0.323, *p* < 0.001) and mothers’ (γ = 0.390, *p* < 0.001) FtoWC was positively associated with their own DASS, supporting H3.

H4 tested partner effects, i.e., FtoWC of one parent is positively associated with the other parent’s (H4a) and the adolescent’s (H4b) DASS. While fathers’ FtoWC was not statistically associated with mothers’ DASS (*γ* = 0.094, *p* = 0.089), mothers’ FtoWC was positively associated with fathers’ DASS (*γ* = 0.154, *p* = 0.004). These findings partially supported H4a. While fathers’ FtoWC was not statistically associated with adolescents’ DASS (*γ* = 0.115, *p* = 0.083), mothers’ FtoWC was positively associated with adolescents’ DASS (*γ* = 0.140, *p* = 0.030). These results supported H4b only for mothers.

H5 tested actor effects for each family member, i.e., DASS is negatively associated with SWFaL for fathers (H5a), mothers (H5b), and adolescents (H5c). Fathers’ (*γ* = −0.359, *p* < 0.001), mothers’ (γ = −0.242, *p* < 0.001), and adolescents’ (*γ* = −0.441, *p* < 0.001) DASS were negatively associated with their SWFaL, therefore supporting H5.

H6 tested partner effects, posing that the DASS of one parent is negatively associated with the other parent’s (H6a) and the adolescent’s (H6b) SWFaL. Fathers’ DASS was not significantly associated with mothers’ SWFaL (*γ* = −0.029, *p* = 0.653). Likewise, mothers’ DASS was not significantly associated with fathers’ SWFaL (γ = 0.005, *p* < 0.935); therefore, H6a was not supported for both parents. Neither fathers’ DASS was significantly associated with adolescents’ SWFaL (γ = −0.066, *p* = 0.335), nor mothers’ DASS was significantly associated with adolescents’ SWFaL (*γ* = 0.078, *p* = 0.206). Therefore, these findings did not support H6b.

Only the adolescents’ age and the fathers’ working hours affected the model significantly as control variables. Adolescents’ age positively affected their DASS (*γ* = 0.136, *p* = 0.008). Fathers’ type of employment (employed vs. self-employed) also positively affected the adolescents’ DASS (*γ* = 0.139, *p* = 0.019); adolescents with self-employed fathers had higher levels of DASS than adolescents with employed fathers.

### 3.3 The moderating role of monthly household income

The monthly household income was examined using multi-group analyses as a categorical variable (≤ USD 1,000 vs. > USD 1,000). The multi-group analysis had fit indices that showed an acceptable fit with the data (RMSEA = 0.066, CFI = 0.997, TLI = 0.897). The monthly household income moderated the association between mothers’ FtoWC and fathers’ DASS (*γ* = −0.270, *p* = 0.015). In families with a monthly household income ≤ USD 1,000, the association between the mothers’ FtoWC was not statistically associated with the fathers’ DASS (*γ* = 0.079, *p* = 0.235). By contrast, in families with a monthly household income > USD 1,000, the association between the mothers’ FtoWC and the fathers’ DASS was stronger (γ = 0.322, *p* < 0.001). The monthly household income also moderated the relationship between mothers’ FtoWC and their SWFaL (γ = −0.601, *p* = 0.005). In families with a monthly household income ≤ USD 1,000, the mothers’ FtoWC and SWFaL relationship was statistically significant (γ = −0.220, *p* = 0.003). The monthly household income also moderated the relationship between mothers’ DASS and their SWFaL (γ = 0.346, *p* = 0.041). In families with a monthly household income ≤ USD 1,000, the association between the mothers’ DASS and SWFaL was weaker (γ = −0.170, *p* = 0.022). By contrast, in families with a monthly household income > USD 1,000, the association between the mothers’ DASS and SWFaL was stronger (γ = −0.386, *p* < 0.001).

### 3.4 The mediating role of psychological distress

Lastly, this study tested the mediating role of the three family members’ DASS between parents’ FtoWC and the three family members’ SWFaL (*Hypothesis 7*, actor and partner effects). The mediating role of the mothers’ DASS in the relationship between her FtoWC and SWFaL was supported (standardized indirect effect = −0.094, 95% CI = −0.256, −0.064, *p* = 0.001). The fathers’ DASS had a mediating role between their FtoWC and SWFaL (standardized indirect effect = −0.116, 95% CI = −0.314, −0.109, *p* < 0.001), and between the mother’s FtoWC and the father’s SWFaL (standardized indirect effect = −0.055, 95% CI = −0.179, −0.023, *p* = 0.011). The adolescents’ DASS mediated between the mothers’ FtoWC and the adolescents’ SWFaL’ (standardized indirect effect = −0.062, 95% CI = −0.257, −0.004, *p* = 0.042). No other mediating role in the three family members’ psychological distress was found. These findings partially supported H7 regarding the mediating role of the three family members’ DASS between parents’ FtoWC and the three family members’ SWFaL.

## 4 Discussion

We examined the relationships between FtoWC, psychological distress, and family satisfaction in different-sex dual-earner parents with adolescents during the second year of the pandemic. Our theoretical basis for this analysis was the COR theory (12) and the matching hypothesis (11). We found that fathers’ FtoWC was directly and negatively related to their family satisfaction and indirectly via their psychological distress. The mothers’ FtoWC was only indirectly and negatively associated with their family satisfaction through their psychological distress. The mothers’ FtoWC was also indirectly and negatively related to the father’s and adolescents’ family satisfaction via these family members’ psychological distress.

### 4.1 Actor effects

We found that higher FtoWC in fathers was linked to lower family satisfaction. This finding partially supports H1, and it aligns with the matching hypothesis (11) and with previous evidence showing that a family-originated conflict is linked to negative outcomes in the family domain (11, 20–23, 30, 31). The null association in mothers suggests that the matching hypothesis is gender-sensitive. Both parents’ FtoWC correlated with increased psychological distress, supporting H3 and consistent with COR theory (15, 27), highlighting the impact of excessive family demands during the COVID-19 pandemic (7, 9, 23, 27, 31, 37, 39, 40).

Higher psychological distress was linked to lower family satisfaction in fathers, mothers, and adolescents (H5 supported). In keeping with the resource loss spiral, our results suggest that individuals’ weakened or depleted personal resources (13, 15) may harm family life and interactions (47) and, ultimately, their family satisfaction, thus making a negative impact on parents and adolescent children. These findings enhance the existing literature on the relationship between psychological distress and family satisfaction in adults (31, 40, 47) and adolescents (48–50).

The negative correlation between psychological distress and family satisfaction in mothers is noteworthy. Schnettler et al. (23) reported that psychological distress was negatively related to job satisfaction only in fathers when studying the interplay between FtoWC, psychological distress, and job satisfaction in dual-earner couples in the first year of the pandemic in Chile. Following the boundary theory (88), these researchers suggested that fathers tend to have a more unified approach to balancing work and family responsibilities. In contrast, mothers tend to separate the two domains, which helps them avoid work-related psychological distress. However, our findings indicate that mothers struggle to prevent their psychological distress from affecting family satisfaction. This novel result may be explained by the gender role theory, which suggests that women closely connect their identities to family roles (76), making them more sensitive to resource loss in this critical domain (10).

### 4.2 Partner effects

We posed that one parent’s FtoWC would be negatively associated with the other parent’s and the adolescent’s family satisfaction. Contrary to expectations (H2a was not supported), our results showed no crossover effect, meaning that fathers’ FtoWC did not affect mothers’ family satisfaction and vice versa. This result contradicts previous findings by Orellana et al. (22) during the first year of the pandemic in Chile. Our outcome may be explained by the fact that when the actor effect is weak or non-significant, such as that shown in fathers and mothers in this study, the partner effect is probably absent (25, 33). However, one potential reason for this finding could be that both parents have similar work circumstances, as most couples in our study were working in person during the second year, reducing their time together (see Table 1). Alternatively, our results could be associated with the possibility that partner effects may be indirect, influenced by other variables (33). This analysis could reflect the psychological distress experienced by fathers, as indicated by Schnettler et al. (23) during the initial year of the pandemic.

Both parents’ FtoWC and the adolescents’ family satisfaction showed no direct relationships (H2b not supported), possibly because adolescents tolerate parents’ inter-role conflict (77). Nevertheless, our findings align with previous studies, showing that parents’ FtoWC is unrelated to their adolescent children’s well-being (17, 34, 35). Other variables may mediate partner effects (33). We proposed psychological distress as a mediating variable between the above relationships for parents and adolescents, as discussed below.

We also proposed that one parent’s FtoWC would be positively associated with the other parent’s and the adolescents’ psychological distress. Findings showed that mothers’ FtoWC positively crossed over to the fathers’ psychological distress but not vice versa (H4a partially supported), aligning with earlier pandemic research (23). This discrepancy may be attributed to heightened gendered expectations during the pandemic (65, 78), suggesting that resource loss in mothers forces them to delegate more household responsibilities to fathers, thereby increasing fathers’ psychological distress.

Moreover, only mothers’ FtoWC crossed over to adolescents (H4b partially supported), increasing their psychological distress, which may also be associated with the higher resource loss experienced by mothers. Previous research shows a connection between parents’ work–family conflict and children’s mental health decline (14, 36, 44–46). Still, our findings suggest that mothers’ FtoWC shapes family dynamics, potentially leading to irritable parenting and fewer relational resources (36, 44). Additionally, adolescents may challenge their parents, creating further conflict that harms their mental well-being (17). Therefore, following Yucel and Borgmann (41), we suggest that these arguments can potentially generate or heighten conflicts between mothers and adolescents, negatively affecting adolescents’ mental health. The lack of a crossover effect from fathers’ FtoWC might be due to traditional gender roles, with mothers more focused on family responsibilities and spending more time at home (79). Thus, their FtoWC affects their children more significantly than fathers’ FtoWC.

Furthermore, we anticipated that one parent’s psychological distress would negatively affect the other parent’s and the adolescent’s family satisfaction. However, we found no evidence for this hypothesis (H6a not supported), as mothers’ distress did not affect fathers’ family satisfaction and vice versa. This finding contradicts the COR theory and previous findings of adverse crossover effects between partners’ psychological distress and family satisfaction (51, 80). Following Westman (33), this lack of crossover may stem from a decline in partners’ empathy after nearly 2 years of the pandemic. Further research is needed to explore these null associations.

Research indicates that depression and stress adversely impact parenting and family interactions, potentially leading to increased conflicts and diminished family satisfaction for children (16, 32, 54, 55). For instance, Orellana, Schnettler, et al. (32) reported that both parents’ mental health was associated with other family members’ family satisfaction. In contrast, we did not find these direct partner effects for either parent (H6b not supported). The pandemic’s influence on work-family dynamics may affect these findings, warranting further research post-pandemic. However, as we discussed in the next section, our results show that psychological distress may be the vehicle that allows indirect partner effects that negatively affect all family members’ family satisfaction.

### 4.3 The mediating effect of psychological distress

We found partial support for the mediating role of psychological distress between parents’ FtoWC and the three family members’ family satisfaction (H7). This mediating role was significant at an intra-individual level regardless of the parent’s gender. Our results support a previous study showing that psychological distress mediates the relationship between FtoWC and family satisfaction in mothers (31). However, they also expand on this knowledge, showing that the same mechanism is found in fathers. At the inter-individual level, the mediating role was found for fathers’ and adolescents’ psychological distress. These results suggest that fathers’ and adolescents’ psychological distress transfer strain from FtoWC to family satisfaction, negatively affecting the family (11). In this regard, FtoWC that involves both parents’ and adolescents’ psychological distress (i.e., stress, anxiety, and depression) can function as a resource loss spiral and, indirectly, negatively affect the domain in which the conflict originates (i.e., family).

Fathers’ psychological distress showed a mediating role between the mothers’ FtoWC and the fathers’ family satisfaction. These findings may be partly explained by the higher FtoWC experienced by mothers during the pandemic (27, 29, 81, 82), which negatively affected not only their family satisfaction but also their partners’. Mothers in our sample scored higher than fathers in the DASS-21, suggesting fathers’ mental health may be more affected by mothers’ FtoWC than the other way around. It should be highlighted that fathers’ psychological distress and family satisfaction have a double negative effect from their and the mothers’ FtoWC.

Lastly, adolescents’ psychological distress showed a mediating role between mothers’ FtoWC and adolescents’ family satisfaction. Previous studies have shown that parent’s negative work experiences can indirectly influence their children’s well-being through negative parent–child relationships (34, 35). Our study contributes to the literature by showing that adolescents’ psychological stress mediates between mothers’ FtoWC and their children’s family satisfaction. These experiences may link to a negative mother-adolescent relationship due to the pandemic’s burden on mothers, potentially increasing adolescents’ psychological distress.

The study also highlights gender patterns. It is noteworthy that mothers’ feelings of being torn between work and family may cause psychological distress for themselves, fathers, and adolescents, as well as affect family satisfaction. This distress could be linked to patriarchal expectations in Latin America that hinder women’s career development, especially for mothers who are expected to prioritize family over work (83). In the Latin American context, men are typically urged to prioritize their jobs, while mothers are expected to manage family-related stressors, such as parenting challenges like children’s misbehavior (84). During the pandemic, men and women faced increased family demands, but men often took on supporting roles while prioritizing their paid work (85). Conversely, women were more responsible for unpaid childcare, schooling, and domestic tasks (34, 85). Thus, greater caregiving and workload burdens have been associated with higher levels of FtoWC, parenting stress, and symptoms of stress, depression, and anxiety in women compared to men [e.g., (27, 29, 82, 83)].

### 4.4 The moderating role of monthly household income

Regarding our research question, we found that the monthly household income moderated three paths in the APIM model, all involving the mothers. The significant association between mothers’ FtoWC and family satisfaction in families with a monthly household income ≤ USD 1,000 suggests that the matching hypothesis is income-sensitive in mothers.

Contrary to the evidence reporting a positive relationship between financial hardships, FtoWC, and mental health problems (2, 9, 26, 59), we obtained that the relationship between mothers’ FtoWC and fathers’ DASS was not significant in families with monthly household income ≤ USD 1,000, while in families with monthly household income > USD 1,000 was stronger. Families with higher incomes may worry about maintaining their living standards, which can increase the risk of mental health issues (26). Thus, it is possible that mothers in this situation may be more concerned about maintaining their levels of FtoWC for longer and transfer this strain to the fathers, negatively affecting their partners’ psychological distress strongly.

During the pandemic in Taiwan, an investigation by Lee and Fung (58) found that clergy earning above the median income had lower anxiety levels than those earning below it. Nevertheless, our results suggest the opposite trend. The association between mothers’ psychological distress and family satisfaction was weaker in families with a monthly household income ≤ USD 1,000, while this relationship was stronger in families with a monthly household income > USD 1,000. Further investigations are needed to understand how household monthly income, particularly in mothers, moderates these findings, which may relate to the Chilean state’s economic measures, including direct transfers and subsidies for lower-income families during the pandemic (86).

### 4.5 Limitations

The study acknowledges its limitations. First, the study design was cross-sectional; thus, the causality cannot be established, and mediation results should be interpreted cautiously. Second, our sample was recruited using a non-probabilistic sampling method and did not represent the Chilean population of dual-earner parents with adolescents because the sample only included families with adolescents between the ages of 10 and 16. Third, the study’s data were collected using self-reported measures, which suggests that participants’ answers may have been influenced by their wish to provide socially acceptable responses. Fourth, the questionnaire asked about the monthly household income, but it did not include questions regarding income loss and economic transference or subsidies that families received during the pandemic. We recommend conducting longitudinal studies with more representative samples and with samples from diverse cultural contexts to better understand family-to-work dynamics and overall work–family conflict after the pandemic. Lastly, the literature will benefit from studies addressing the experiences of work-to-family conflict and family-to-work conflict, taking the cross-domain relationship (87) and the matching hypothesis (11), respectively.

The strength of this study lies in its large sample size, which surveyed 430 mothers-father-adolescent triads (i.e., 1,290 individuals). In addition, the APIM approach makes it possible to analyze three family members simultaneously, taking both parents’ FtoWC and the three family members’ psychological distress and family satisfaction. This approach provides a valuable overview of the interdependence between family members and their living conditions and well-being.

## 5 Implications and conclusion

Our findings contribute to the work-family interface literature by showing the potential associations between FtoWC, psychological distress, and family satisfaction in mother–father-adolescent triads. Fathers’ FtoWC negatively influences their family satisfaction directly and via psychological distress. By contrast, the mothers’ FtoWC indirectly influences their, the fathers’, and adolescents’ family satisfaction via each family member’s psychological distress.

Due to our research being conducted on a sample of dual-earner parents with adolescent children, most of whom worked in-person, our findings remain relevant for future public health crises and families’ experiences in the post-COVID era. Our findings underscore the need for further research to explore family-to-work conflict after the pandemic. Family-to-work conflict might be maintained and moderated by work conditions established in response to the pandemic. We also propose further studying the conditions of different-sex dual-earner parents with adolescents and considering that FtoWC outcomes may be triadic. Future research should examine whether parents’ psychological distress leads to hostile family environments and parenting practices that harm children’s well-being, given that work–family conflict negatively affects family dynamics. In addition, future research should study the conditions in which the matching hypothesis is gender-sensitive or not and income-sensitive in mothers. Proposed studies should include families of various sizes and compositions, examining their relationship to traditional versus egalitarian gender roles to better understand family-to-work experiences.

These findings also entail practical implications. Our findings highlight the importance of workplace policies and training to help workers, especially mothers, manage their various life roles. Stress management resources and family-friendly policies are essential to improving workers’ well-being and satisfaction. Interventions require a gender perspective to address men’s and women’s demands in work and family life. These interventions should highlight men’s multiple roles to promote their involvement at home and help reduce work–family conflict for women.

## Data Availability

The raw data supporting the conclusions of this article will be made available by the authors, without undue reservation.
